# Factor XIa and Thrombin Generation Are Elevated in Patients with Acute Coronary Syndrome and Predict Recurrent Cardiovascular Events

**DOI:** 10.1371/journal.pone.0158355

**Published:** 2016-07-15

**Authors:** Rinske Loeffen, René van Oerle, Mathie P. G. Leers, Johannes A. Kragten, Harry Crijns, Henri M. H. Spronk, Hugo ten Cate

**Affiliations:** 1 Laboratory for Clinical Thrombosis and Haemostasis, Departments of Internal Medicine and Biochemistry, Cardiovascular Research Institute Maastricht, Maastricht University Medical Center, Maastricht, The Netherlands; 2 Departments of Clinical Chemistry & Hematology, Atrium Medical Center Parkstad, Heerlen, The Netherlands; 3 Department Of Cardiology, Atrium Medical Center Parkstad, Heerlen, The Netherlands; 4 Department of Cardiology, Cardiovascular Research Institute Maastricht, Maastricht University Medical Center, Maastricht, The Netherlands; University Hospital Medical Centre, GERMANY

## Abstract

**Objective:**

In acute coronary syndrome (ACS) cardiac cell damage is preceded by thrombosis. Therefore, plasma coagulation markers may have additional diagnostic relevance in ACS. By using novel coagulation assays this study aims to gain more insight into the relationship between the coagulation system and ACS.

**Methods:**

We measured plasma thrombin generation, factor XIa and D-dimer levels in plasma from ACS (n = 104) and non-ACS patients (n = 42). Follow-up measurements (n = 73) were performed at 1 and 6 months. Associations between coagulation markers and recurrent cardiovascular events were calculated by logistic regression analysis.

**Results:**

Thrombin generation was significantly enhanced in ACS compared to non-ACS patients: peak height 148±53 vs. 122±42 nM. There was a significantly diminished ETP reduction (32 vs. 41%) and increased intrinsic coagulation activation (25 vs. 7%) in ACS compared to non-ACS patients. Furthermore, compared to non-ACS patients factor XIa and D-dimer levels were significantly elevated in ACS patients: 1.9±1.1 vs. 1.4±0.7 pM and 495(310–885) vs. 380(235–540) μg/L. Within the ACS spectrum, ST-elevated myocardial infarction patients had the highest prothrombotic profile. During the acute event, thrombin generation was significantly increased compared to 1 and 6 months afterwards: peak height 145±52 vs. 100±44 vs. 98±33 nM. Both peak height and factor XIa levels on admission predicted recurrent cardiovascular events (OR: 4.9 [95%CI 1.2–20.9] and 4.5 [1.1–18.9]).

**Conclusion:**

ACS patients had an enhanced prothrombotic profile, demonstrated by an increased thrombin generation potential, factor XIa and D-dimer levels. This study is the first to demonstrate the positive association between factor XIa, thrombin generation and recurrent cardiovascular events.

## Introduction

Thrombotic occlusion of a coronary artery resulting in myocardial underperfusion is the key event in the development of the acute coronary syndrome (ACS) [1, 2]. The principal cause is the rupture of a coronary atherosclerotic plaque, which triggers thrombosis through the activation of the hemostatic system [3, 4]. According to Virchow’s triad, the risk of atherothrombosis is depending on plaque vulnerability, shear stress, and systemic factors in the circulating blood (i.e. hemostatic and cellular blood components) [5]. Besides the well-established role of platelets [6], there is substantial evidence suggesting the involvement of the coagulation system in the pathogenesis of ACS [7, 8]. Several studies showed that during the acute phase of ACS the coagulation system is activated and elevated levels of markers of activated coagulation, such as thrombin-antithrombin complexes (TAT), prothrombin fragment 1.2 and D-dimer, have been demonstrated in those patients [9–13]. Moreover, a persistent hypercoagulable state after clinical stabilization has also been demonstrated in ACS patients [10]. However, whether increased coagulation is the cause or consequence of thrombotic events remains difficult to establish in clinical research.

The spectrum of ACS is divided in ST-segment elevation myocardial infarction (STEMI), non-ST-segment elevation myocardial infarction (NSTEMI) and unstable angina (UA). Subdivision of patients is based on electrocardiogram (ECG) and biomarkers of cardiomyocyte necrosis [14]. Since cardiac cell damage is preceded by thrombosis, plasma coagulation markers may have additional diagnostic relevance in ACS. However, in spite of major achievements during the last decades to define the relationship between coagulation and ACS there is still need for new and improved diagnostic approaches to better understand the relationship between hypercoagulability and ACS. Therefore, this study aims to gain more insight into the role of coagulation markers in ACS. By using both novel (assays for free factor XIa and for factor Xa- and factor IXa-antithrombin complexes) and existing coagulation assays we investigated differences in coagulability between UA, NSTEMI and STEMI and non-ACS patients, both during the acute phase and after clinical stabilization.

## Methods

### Study design & population

We performed an exploratory prospective cohort study including ACS patients at an early stage of the coronary ischemic event. Inclusion criteria: eligible patients presented with chest pain suspect for ACS in the ambulance between April 2012 and September 2013, and were transported to the Maastricht University Medical Center or Atrium MC Hospital Heerlen. Patients were included when diagnosed with ACS during admission. The diagnosis of ACS was based on electrocardiogram (ECG) and biomarkers of cardiomyocyte necrosis according to the international criteria [14]. Patients who were admitted for ischemia detection and were diagnosed with ACS after admission were also included in the ACS cohort. Blood collected from ACS negative (non-ACS) patients with identical pre-analytical procedures served as a reference population. Non-ACS patients, who did not meet the ACS criteria, were transported by ambulance with suspected cardiac pain; they had no ECG or laboratory changes indicative of myocardial damage and were discharged from the hospital without a cardiological diagnosis and follow-up. Because of possible undiagnosed underlying coronary problems, all patients that required further cardiological analysis (e.g. ischemia detection) during follow-up appointments at the cardiology department or outpatient clinic were not included as non-ACS patients.

Subjects using oral anticoagulants were excluded. Blood samples were collected in the ambulance, before administration of low-molecular-weight heparin (LMWH) or any other intervention, and subsequently 1 month and 6 months after inclusion at one of the participating hospitals. Venous blood was collected from the antecubital vein using 21-gauge needles and Venoject 3.6 ml, 3.2% (w/v) citrated tubes (Terumo Europe, Leuven, Belgium). Platelet-poor plasma was obtained within 90 min by two separate centrifugation steps of 5 min at 2000 x *g*, followed by 10 min at 11.000 x *g* and plasma aliquots were stored at -80°C until analysis.

Clinical outcome was recorded 1 and 6 months after inclusion and comprised the combined endpoint of cardiovascular death, recurrent MI, a second coronary intervention (percutaneous coronary intervention or coronary artery bypass grafting), and ischemic stroke. All participants, both ACS and non-ACS provided written informed consent and the study was conducted according to the principles of the Declaration of Helsinki and the laws and regulations applicable in the Netherlands. The local institutional review board (METC azM/UM) approved the conduct of this study.

### Laboratory assays

#### Thrombin generation

Thrombin generation in platelet-poor plasma was measured by means of the Calibrated Automated Thrombogram (CAT) method (Thrombinoscope BV, Maastricht, The Netherlands), according to our previously described standardized protocol [15]. The CAT method is an in vitro plasma assay that reflects the overall tendency of a plasma sample to clot and provides a quantification of the total amount of thrombin formed.

Coagulation was activated by the addition of tissue factor (TF), phospholipids (PL) and calcium chloride. Thrombin activity was monitored via the conversion of a low-affinity fluorogenic substrate for thrombin added to the plasma. To obtain information about the different coagulation pathways in ACS patients, thrombin generation measurements were performed under different experimental conditions. Measurements were performed with addition of 0 and 1 pM TF together with 4 μM PL (All CAT-reagents were obtained from Thrombinoscope BV). To investigate the contribution of the intrinsic coagulation pathway, measurements were conducted in the absence of TF and with the addition of the extrinsic pathway inhibitor active site-inhibited factor VIIa (ASIS, Novo Nordisk, Bagsvaerd, Denmark) in a final concentration of 30 nM and with the addition of corn trypsin inhibitor (CTI) to inhibit factor XIIa, at a final concentration of 25 μg/ml. Furthermore, to investigate the function of the protein C pathway 0.65 nM (final concentration) recombinant soluble thrombomodulin (Thrombinoscope BV) was added to the 1 pM TF measurements, sufficient to inhibit thrombin generation in platelet-poor normal pooled plasma by 50%. Normal pooled plasma was collected at the Hematology Department of the Maastricht University Medical Center (Maastricht, The Netherlands), by pooling platelet poor plasma from 80 healthy volunteers without using any medication.

Measurements were performed after 10 minutes (min) of preheating at 37°C in the fluorometer. Fluorescence was read in a Fluoroskan Ascent reader (Thermo Labsystems OY, Helsinki, Finland). The following parameters were derived from the thrombin generation curves: endogenous thrombin potential (ETP, area under the curve), time to peak, peak height (maximum amount of thrombin formed), and the velocity index (slope).

#### Factor XIa CAT assay

Plasma levels of activated factor XI were assessed using the CAT method, under adjusted testing conditions, as previously described [16]. In the factor XIa CAT assay all measurements were conducted in the absence of TF and activation was only by addition of 4 μM PL. To inhibit the extrinsic pathway, ASIS was added at a final concentration of 30 nM. All plasma samples were diluted at a 1:5 ratio in factor XI deficient plasma (Haematologic Technologies Inc., Essex Junction, VT, USA).

Factor XIa plasma levels were calculated from a reference curve developed with eight sequential dilutions of purified human factor XIa (0–12.5 pM; Haematologic Technologies Inc.) in a plasma mixture of pooled corn trypsin inhibitor (CTI) plasma and factor XI deficient plasma. CTI plasma from 30 healthy volunteers (Maastricht University Medical Center, The Netherlands) was obtained using SCAT 4.5 ml, 50 μg mL^-1^ CTI/3.2% (w/v) citrate tubes (Haematologic Technologies Inc.), followed by two-step centrifugation (5 min at 2000 x *g*, followed by 10 min at 11.000 x *g*) and pooling of platelet-poor plasmas.

Patients’ citrated plasma samples were diluted in the same manner in factor XI deficient plasma (1:5 ratio), with addition of CTI at a final concentration of 25 μg/ml, to inhibit factor XIIa and analyzed in duplicate. As previously described, all patients’ plasma samples were measured in the absence and presence of an inhibitory monoclonal antibody directed against factor XIa (final concentration of 100 nM), to establish that the factor XIa plasma level was the only determinant of the thrombin generation curve [16].

#### D-dimer and thrombin-antithrombin (TAT) complexes

Plasma concentrations of D-dimer were established using a Sysmex^®^ CA-7000 System Automated Coagulation Analyzer with reagents obtained from Siemens Healthcare Diagnostics (Marburg, Germany). D-dimer measurements were performed using the INNOVANCE^®^ D-dimer assay [Reference range: < 550 ng/mL]. TAT complexes were quantified by a commercially available immunoassay (Enzygnost^®^ TAT Micro, Siemens Healthcare Diagnostics, Marburg, Germany) [Reference range: 2.0–4.2 ng/mL].

#### Antithrombin (AT)-inhibitor complexes assays

Factor IXa-AT and factor Xa-AT in-house enzyme-linked immunosorbent assays (ELISAs) were developed to establish AT-inhibitor complexes levels. Sheep anti-human factor IX or X (Affinity Biologicals Inc., Ancaster, Canada) at a final concentration of 2 μg/ml was added to Nunc maxisorp immunoplates (Thermo Fisher Scientific Inc., Waltham, MA, USA) and incubated overnight at 4°C. The plates were washed with washing buffer followed by blocking of the plates for 2 hours at 37°C using a blocking solution (200 μL/well). Subsequently, plasma samples diluted in blocking buffer were added to the plate together with established control samples (factor IX-AT or factor X-AT) and incubated at 300 rpm for 2 hours at room temperature (RT). Following incubation the plates were washed and incubated with a biotin-labeled sheep anti-human antithrombin detection antibody (100 μl/well) (Haematologic Technologies Inc.) for 1 hour at RT. After a subsequent washing step, the plates were incubated with Avidin-Horseradish Peroxidase (Affinity Biologicals Inc. Ancaster, ON Canada) for 30 min, followed by washing of the plates and incubation for 30 min with the substrate solution (TMB Peroxidase Substrate, Lucron Bioproducts, Gennep, The Netherlands). The absorbance was measured at 450 nm using a microplate reader (Bio-Rad Laboratories BV, Veenendaal, the Netherlands). Reference range for factor IXa-AT: 78–215 pM and for factor Xa-AT: 354–766 pM.

### Statistical Analysis

Statistical analysis was performed with PRISM for Mac, version 6.00 (GraphPad Software, San Diego, CA, USA), and SPSS version 21.0 (SPSS inc. Chicago, IL, USA). According to the distribution of variables data are expressed as mean ± standard deviation (SD) or median ± interquartile range (IQR). Categorical variables are expressed as number with percentage (%). Differences between two groups were analyzed using a chi-square test (χ²), unpaired Student’s t-test or Mann-Whitney U test as appropriate. A one-way ANOVA or Kruskal-Wallis test with multiple comparison analysis was used for analysis of differences between more than two groups, and a Repeated measured ANOVA or Friedman test for establishing the differences between baseline and follow-up measurements. Nonlinear regression analysis of the reference curve peak height values, using a hyperbola curve fit, was used to obtain the factor XIa concentrations of the plasma samples. A two-tailed probability value of P<0.05 was considered statistically significant. Thrombin generation parameters, factor XIa, D-dimer and antithrombin complexes levels were categorized in quartiles and the associations between coagulation markers and outcome were assessed by logistic regression analysis with either the highest or the lowest quartile as reference category. Odds ratios (ORs) are presented with 95% confidence intervals (CIs).

## Results

In total, 104 patients diagnosed with ACS were enrolled in the study, of which 73 ACS patients signed informed consent for follow-up appointments at 1 and 6 months. These ACS patients were compared to 42 non-ACS patients, who did not meet the ACS criteria and were discharged from the hospital without (cardiological) follow-up. Table 1 presents the baseline characteristics for ACS baseline, non-ACS and ACS follow-up patients. There were no significant differences in baseline variables between the total ACS cohort and the subgroup that completed the 6 months’ follow-up. The mean age on admission in the ACS cohort was 67 years (range 42–100) and 68% (n = 71) were male. The group of non-ACS patients comprised 55% males (n = 23) with a mean age of 62 years (range 31–88) and was significantly younger than the ACS cohort (*p* = 0.035, Table 1). The distribution of classical cardiovascular risk factors was similar between ACS and non-ACS patients. Also, there was no significant difference in the distribution of cardiovascular medication between ACS and non-ACS patients. As expected, there were important differences in cardiac markers, with increased troponins, creatinine kinase (CK) and aspartate aminotransferase (ASAT) in the ACS patients compared to the non-ACS patients (*p*<0.0001, Table 1). Furthermore, at baseline glucose and CRP levels were significantly higher in ACS versus non-ACS patients.

**Table 1:** Baseline characteristics of ACS baseline, ACS follow-up and non-ACS patients. ACS, acute coronary syndrome; FU, follow-up; BMI, Body Mass Index; CAD, coronary artery disease; PCI, percutaneous coronary intervention; CABG, coronary artery bypass graft; TIA, transient ischemic attack, ACE, angiotensin-converting enzyme; ATII, angiotensin II receptor antagonist; TnT, Troponin T; hsTnT, high-sensitivity Troponin T; CK, creatinine kinase; LDH, lactate dehydrogenase; ASAT, aspartate aminotransferase; CRP, C-reactive protein. Continuous data are presented as mean ± SD or median ± IQR and statistical analysis of continuous data was performed using unpaired Student’s t-test or Mann-Whitney U test. Categorical variables are presented as %, with differences between the groups tested with χ². P-value^1^: ACS baseline vs. non-ACS patients; P-value^2^: ACS baseline vs. ACS follow-up patients.

	ACS Baseline (n = 104)	Non-ACS (n = 42)	ACS Follow-up (n = 73)	P-value^1^	P-value^2^
Age, yrs (range)	67 (42–100)	62 (31–88)	66 (42–100)	0.035	0.744
Sex, m (%)	71 (68.3)	23 (54.8)	54 (74.0)	0.129	0.412
BMI, kg/m^2^	26.3 (24.2–29.1)	28.0 (26.0–30.9)	26.3 (24.2–29.4)	0.169	0.970
Smoking status, n (%)					
Current	20 (19.2)	8 (19.0)	14 (9.2)	0.979	0.993
Former	59 (56.7)	20 (47.6)	48 (65.8)	0.317	0.227
Family history of CAD	39 (37.5)	15 (35.7)	29 (35.7)	0.839	0.764
**Medical history**					
Hypertension, n (%)	44 (42.3)	17 (40.5)	26 (35.6)	0.839	0.370
Diabetes type II, n (%)	16 (15.4)	4 (9.5)	11 (15.1)	0.351	0.954
Hypercholesterolemia, n (%)	43 (41.3)	16 (38.1)	27 (37.0)	0.717	0.559
Myocardial infarction, n (%)	18 (17.3)	7 (16.7)	13 (17.8)	0.926	0.931
PCI, n (%)	18 (17.3)	9 (21.4)	13 (17.8)	0.562	0.931
CABG, n (%)	10 (9.6)	2 (4.8)	9 (12.3)	0.334	0.566
Claudication, n (%)	2 (1.9)	0 (0.0)	2 (2.7)	0.366	0.719
Stroke/TIA, n (%)	8 (7.7)	3 (7.1)	5 (6.8)	0.909	0.832
**Medication**					
Platelet inhibitors					
Aspirin	39 (37.5)	15 (35.7)	26 (35.6)	0.839	0.798
Clopidogrel	8 (7.7)	3 (7.1)	6 (8.2)	0.909	0.898
Dipyridamole	2 (1.9)	1 (2.4)	1 (1.4)	0.859	0.779
Antihypertensive agents					
ACE-inhibitor	10 (9.6)	7 (16.7)	9 (12.3)	0.229	0.566
ATII-antagonist	16 (15.4)	2 (4.8)	10 (13.7)	0.077	0.755
Calcium antagonist	16 (15.4)	6 (14.3)	12 (16.4)	0.867	0.850
Diuretic	18 (17.3)	6 (14.3)	11 (15.0)	0.656	0.692
Nitrate	15 (14.4)	5 (11.9)	10 (13.7)	0.689	0.892
β-blocker	32 (30.8)	9 (21.4)	20 (27.4)	0.256	0.628
Statins	41 (39.4)	15 (35.7)	26 (35.6)	0.677	0.607
**Laboratory measures**					
Troponin					
TnT (0.01–0.1 μg/L)	0.87 (0.03–6.72)	0.0 (0.0–0.0)	1.21 (0.06–9.60)	<0.0001	0.456
hsTnT (3–14 ng/L)	42 (16–255)	8.0 (4.0–12.0)	42 (16–228)	<0.0001	0.959
CK (♂<225, ♀<160 U/L)	179 (97–453)	95 (62–142)	177 (80–445)	<0.0001	0.548
LDH (<480 U/L)	211 (171–258)	187 (163–242)	211 (172–245)	0.256	0.924
ASAT (<35 U/L)	35 (25–75)	24 (20–33)	36 (25–75)	<0.0001	0.828
CRP (<10 mg/L)	4.0 (2.0–9.0)	2.0 (0.0–2.0)	4.0 (2.0–8.3)	0.002	0.943
Glucose (<7.8 mmol/L)	7.0 (6.1–8.6)	6.3 (5.4–6.7)	6.9 (6.0–8.3)	0.002	0.593
Creatinine (♂<110, ♀<95 μmol/L)	76 (66–86)	79 (65–92)	76 (68–87)	0.753	0.750

The ACS group consisted of 58 patients that suffered from STEMI, 24 from NSTEMI and 22 from UA. There were no differences in age and sex between the subgroups (data not shown). However, significantly less STEMI patients were smoking, had a cardiovascular history and were on medication (platelet inhibitors, anti-hypertensive agents or statins).

### Thrombin generation potential

The results for the baseline measurements for the total ACS cohort and for the three subgroups are shown in Table 2. Overall, the thrombin generation potential was enhanced in ACS compared to non-ACS patients. At 1 pM TF (Table 2), ACS patients had a significantly shorter time to peak and time to tail (*p* = 0.002, *p* = 0.01, respectively), a higher velocity index and an increased peak height (*p* = 0.001, *p* = 0.003, respectively). Furthermore, the inhibition of thrombin generation by addition of thrombomodulin resulted in a decreased ETP reduction (<50%) in all groups. Compared to the non-ACS group, there was a significantly diminished ETP reduction in the total ACS group (*p*<0.0001).

**Table 2:** Baseline levels of coagulation activation markers in ACS and non-ACS patients. TG, thrombin generation; TF, tissue factor; ETP, endogenous thrombin potential; TAT, thrombin-antithrombin complexes; AT, antithrombin; ACS, acute coronary syndrome; STEMI, ST-segment elevation myocardial infarction; NSTEMI, non-ST-segment elevation myocardial infarction; UA, unstable angina. Data are presented as mean ± SD or median ± IQR.

	Total ACS	STEMI	NSTEMI	UA	Non-ACS
	(n = 104)	(n = 58)	(n = 24)	(n = 22)	(n = 42)
TG at 1 pM TF trigger					
time to peak (min)	9.6 (1.6)	9.5 (1.6)	9.8 (1.3)	9.6 (1.8)	10.5 (1.5)
peak height (%)	148 (53)	151 (52)	137 (48)	149 (60)	122 (42)
ETP (nM.min)	1161 (268)	1175 (279)	1135 (272)	1152 (239)	1085 (246)
velocity index (nM/min)	30 (22–43)	32 (24–43)	28 (20–41)	27 (19–48)	22 (16–31)
ETP reduction (%)	32 (14)	30 (14)	35 (14)	32 (16)	41 (10)
Factor XIa (pM)	1.9 (1.1)	1.8 (0.9)	2.2 (1.3)	1.6 (0.9)	1.4 (0.7)
D-dimer (μg/mL)	495 (310–885)	510 (283–1148)	560 (345–838)	375 (290–968)	380 (235–540)
TAT (μg/L)	5.1 (3.2–13.6)	5.1 (3.5–14.9)	4.4 (3.1–6.9)	5.1 (2.8–18.0)	4.6 (3.1–7.7)
Factor IXa-AT (pM)	149 (118–194)	149 (120–208)	143 (112–183)	154 (109–218)	144 (112–178)
Factor Xa-AT (pM)	320 (264–408)	337 (287–413)	272 (242–380)	306 (262–395)	334 (292–385)

**Fig 1:**
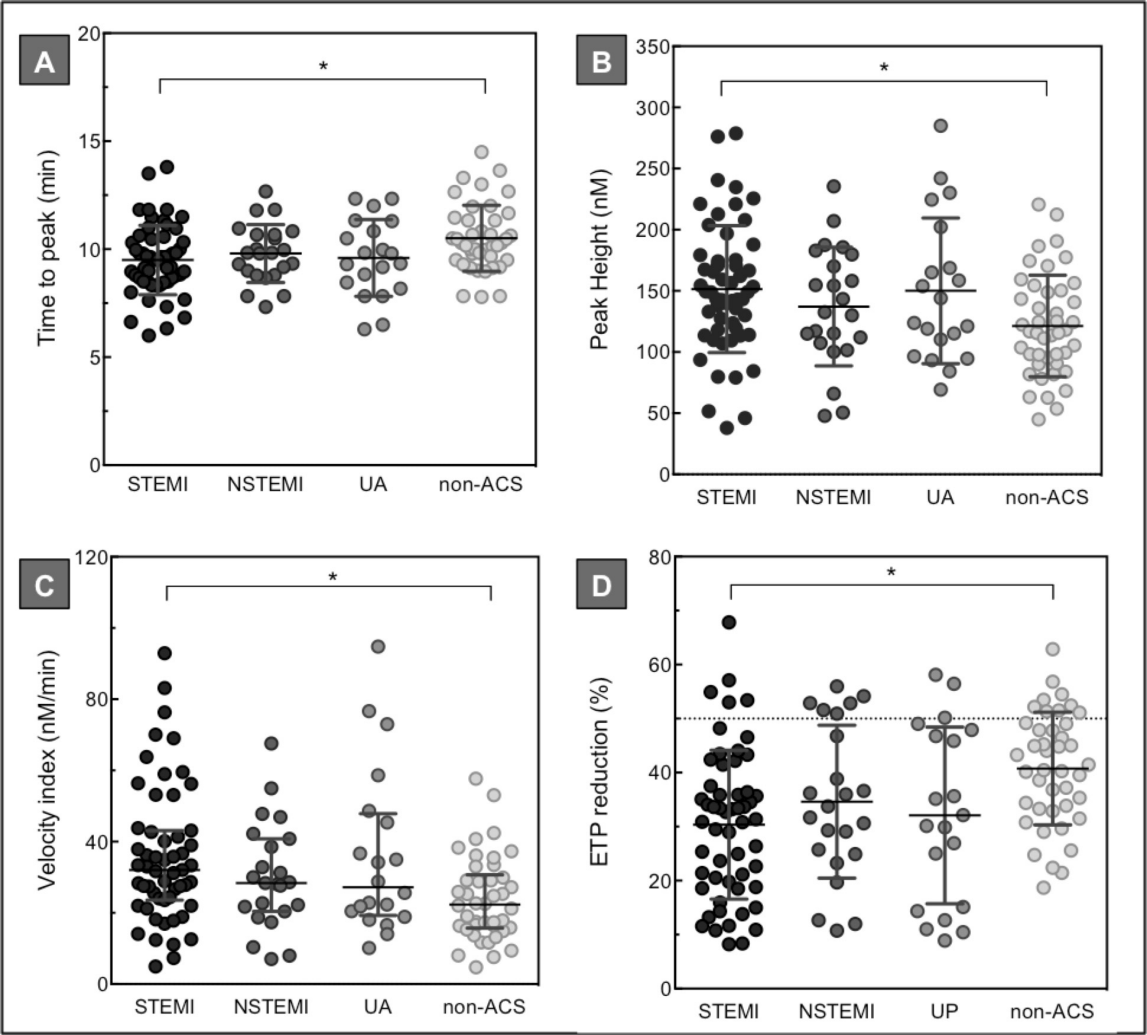
Baseline plasma thrombin generation levels in ACS versus non-ACS patients. Thrombin generation analysis (1 pM TF) of plasma collected from 104 ACS and 42 non-ACS patients during the acute event. Upper panel: time to peak in min (A), and peak height in nM (B). Lower panel: velocity index in nM/min (C), and ETP reduction in % (D). Panel D represents thrombin generation analysis upon addition of thrombomodulin titrated at an ETP reduction of 50% in normal pooled plasma (horizontal dotted line). Depicted are the percentage reductions in ETP. Horizontal lines indicate means ± SD (A, B, D), or medians ± IQR (C). Differences between the groups were tested with a one-way ANOVA or Kruskal-Wallis test with multiple comparison analysis. * *p*<0.05.

The results of the 1 pM TF thrombin generation measurements for the ACS subgroups (STEMI, NSTEMI, and UP) during the acute thrombotic event are depicted in Fig 1. As shown, at the time of the coronary event, the potential to generate thrombin was most enhanced in the STEMI group. Compared to non-ACS patients, STEMI patients had a shorter time to peak (mean 10.5 ± 1.5 min vs. 9.5 ± 1.6 min, *p* = 0.016) and an increased peak height (mean 122 ± 42 nM vs. 151 ± 52 nM, p = 0.02) and velocity index (median 32 [24–43] nM/min vs. 22 [16–31] nM/min, *p* = 0.008). Also, compared to non-ACS patients, STEMI patients showed a significantly lower ETP reduction upon thrombomodulin addition (mean 41 ± 10% vs. 30 ± 14%, *p* = 0.002).

To investigate the contribution of the intrinsic coagulation pathway, measurements were conducted in the absence of TF and with the addition of the extrinsic pathway inhibitor ASIS and the intrinsic pathway inhibitor CTI. We observed that intrinsic coagulation activation was present in significantly more ACS patients than non-ACS patients (25% vs. 7%). In the ACS subgroups, contact activation was highest in STEMI patients, with 31% of STEMI, 22% of NSTEMI and 20% of UP patients showing a thrombin generation curve after addition of both ASIS and CTI to the plasma (data not shown).

### Coagulation activation markers

**Fig 2:**
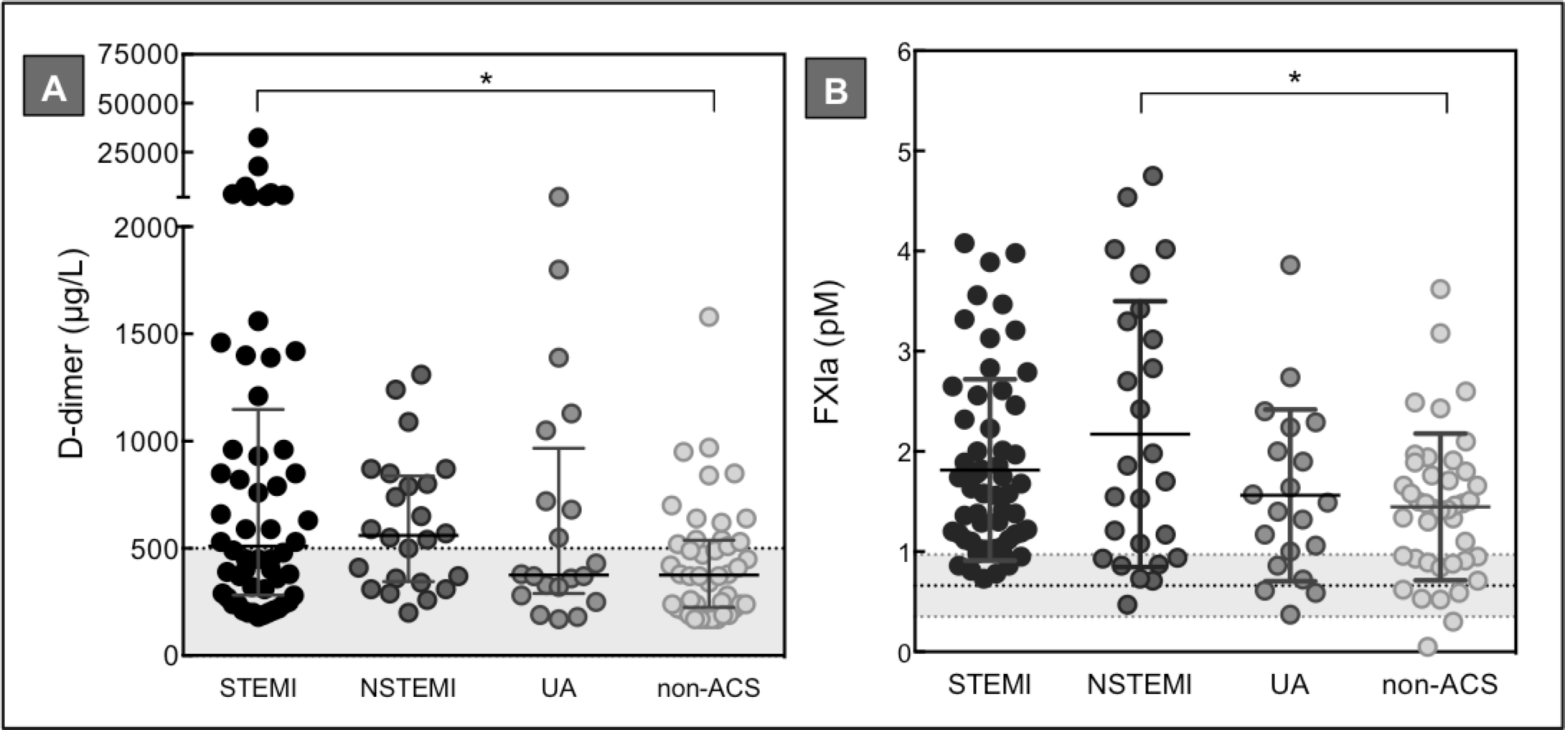
Baseline factor XIa and D-dimer levels in ACS versus non-ACS patients. D-dimer (A) and factor XIa (B) measurements of plasma collected from 104 ACS and 42 non-ACS patients. The horizontal dotted lines represent the D-dimer cut-off value of 500 μg/L (A) and the mean factor XIa level ± SD in a group of healthy individuals, n = 30 (B). Horizontal lines indicate means ± SD (B), or medians ± IQR (A). Differences between groups were tested with a one-way ANOVA or Kruskal-Wallis test with multiple comparison analysis. * *p*<0.05.

At the time of the coronary event, median D-dimer levels were significantly higher in the total ACS cohort than in the non-ACS patients: 495 (310–885) μg/L vs. 380 (235–540 μg/L), *p* = 0.009 (Table 2). Both in STEMI and NSTEMI patients, median D-dimer levels were above the cut-off value of 500 μg/L (Fig 2A), which indicates that in a large subgroup of ACS patients active coagulation was ongoing. Multiple comparison analysis revealed that only in STEMI patients D-dimer levels were significantly higher than in non-ACS patients (median 510 [283–1148] μg/L vs. 380 [235–540] μg/L, *p* = 0.02).

In both ACS and non-ACS patients median TAT levels were above the normal reference range of 4.2 μg/L (Table 2). However, there was no significant difference in TAT levels between ACS and non-ACS patients or between the ACS subgroups and non-ACS patients.

Regarding plasma factor XIa levels, we observed that at the time of the acute event factor XIa plasma levels were significantly elevated in ACS compared to non-ACS patients (mean 1.9 ± 1.1 pM vs. 1.4 ± 0.7 pM, *p* = 0.006) (Table 2). The factor XIa reference range, established by measuring factor XIa plasma levels in 30 healthy individuals, was set between 0.35–0.97 pM. As shown in Fig 2B, in all groups, the mean factor XIa plasma level was above the reference range, with the highest level in NSTEMI patients. Compared to the non-ACS patients, the factor XIa plasma level was significantly higher in NSTEMI patients (mean 1.4 ± 0.7 pM vs. 2.2 ± 1.3 pM, *p* = 0.012).

Results of the two ELISAs developed to establish inhibitor complexes levels of factor IXa-AT and factor Xa-AT revealed comparable inhibitory complexes levels between ACS and non-ACS patients (Table 3).

**Table 3:** Levels of coagulation activation markers in ACS patients during follow-up. TG, thrombin generation; TF, tissue factor; ETP, endogenous thrombin potential; ACS, acute coronary syndrome; STEMI, ST-segment elevation myocardial infarction; NSTEMI, non-ST-segment elevation myocardial infarction; UA, unstable angina. Data are presented as mean ± SD or median ± IQR.

ACS follow-up patients (n = 73)	Acute event	1 month	6 months
TG at 1 pM TF trigger			
time to peak (min)	9.7 (1.7)	10.2 (1.6)	9.8 (1.4)
peak height (nM)	145 (52)	100 (44)	98 (33)
ETP (nM.min)	1147 (264)	872 (307)	845 (246)
velocity index (nM/min)	29 (22–43)	20 (13–26)	19 (14–25)
ETP reduction (%)	33 (15)	38 (14)	42 (12)
Factor XIa (pM)	1.9 (1.1)	1.7 (0.9)	2.2 (1.1)
D-dimer (μg/mL)	400 (270–800)	420 (250–920)	340 (240–630)

### Follow-up

**Fig 3:**
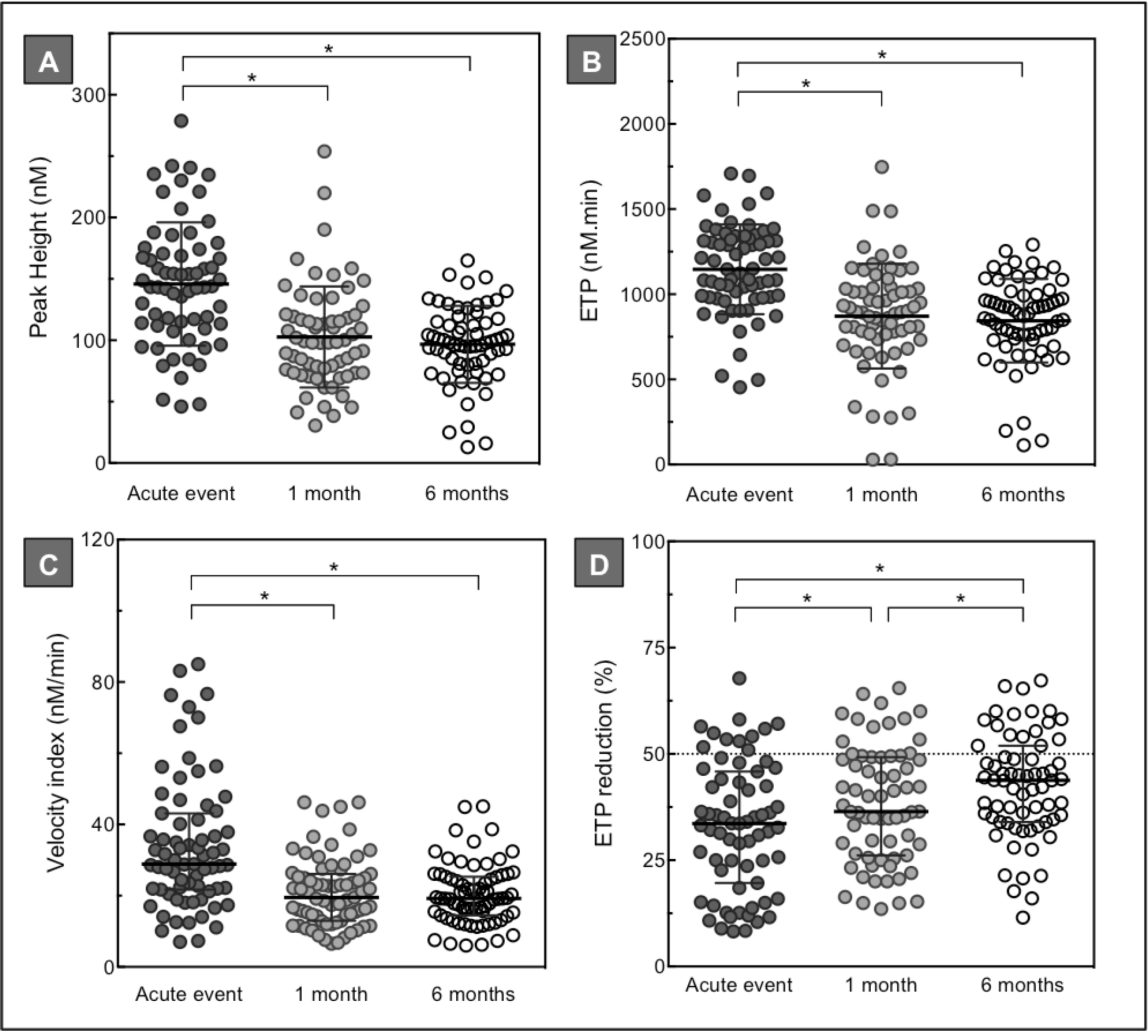
Plasma thrombin generation levels in ACS patients during follow-up. Thrombin generation analysis (1 pM TF) of plasma collected from 73 ACS patients during the acute event and after 1 and 6 months follow-up. Upper panel: peak height in nM (A), and ETP in nM.min (B). Lower panel: velocity index in nM/min (C), and ETP reduction in % (D). Panel D represents thrombin generation analysis upon addition of thrombomodulin titrated at an ETP reduction of 50% in normal pooled plasma (horizontal dotted line). Depicted are the percentage reductions in ETP. Horizontal lines indicate means ± SD (A, B, D), or medians ± IQR (C). Differences between baseline and follow-up measurements were established by repeated measured ANOVA or Friedman test with multiple comparison analysis. * *p*<0.05.

**Fig 4:**
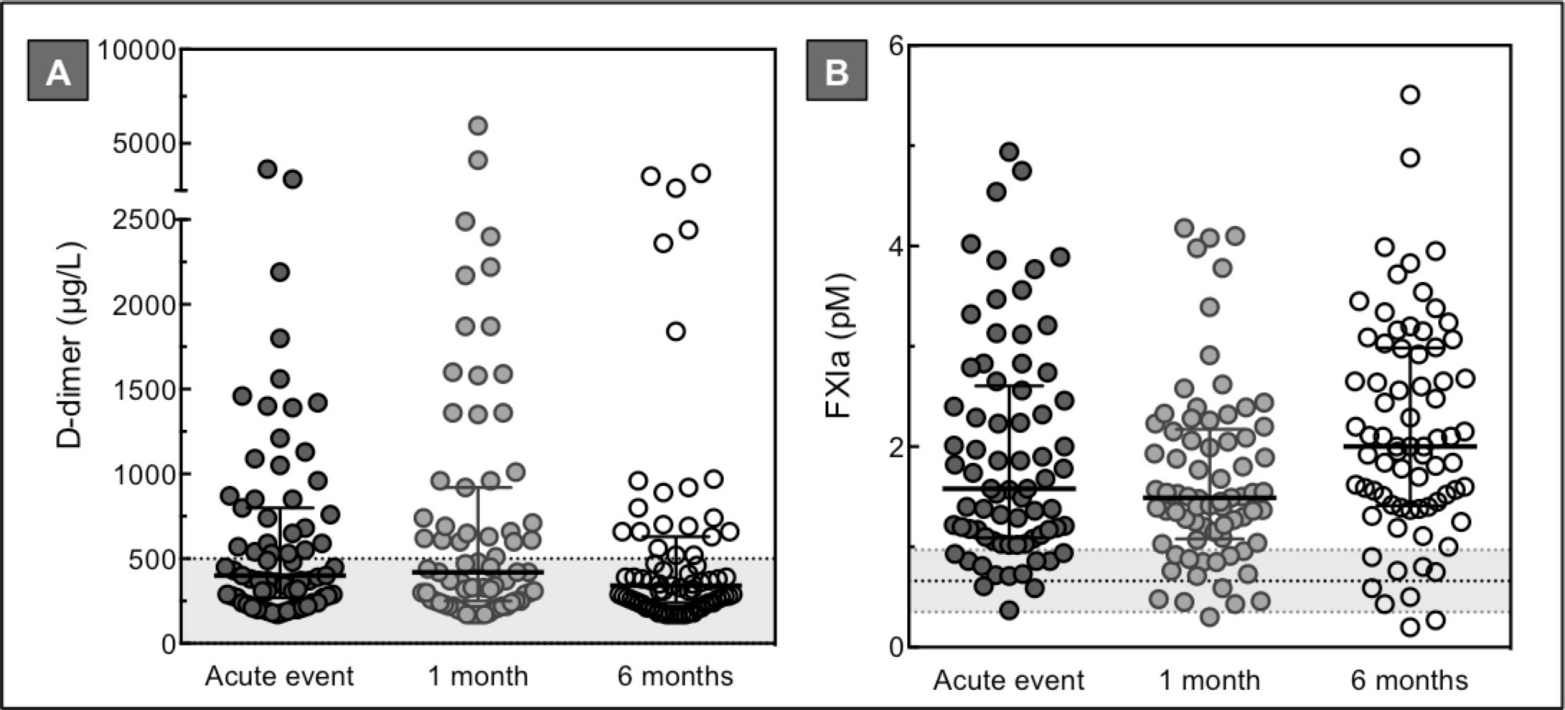
Factor XIa and D-dimer levels in ACS patients during follow-up. D-dimer (A) and factor XIa (B) measurements of plasma collected from 73 ACS patients during the acute event and after 1 and 6 months follow-up. The horizontal dotted lines represent the D-dimer cut-off value of 500 μg/L (A) and the mean factor XIa level ± SD in a group of healthy individuals, n = 30 (B). Horizontal lines indicate means ± SD (B), or medians ± IQR (A). Differences between baseline and follow-up measurements were established by repeated measured ANOVA or Friedman test with multiple comparison analysis. * *p*<0.05.

In total, 73 patients diagnosed with ACS signed informed consent for follow-up appointments, consisting of 41 STEMI, 15 NSTEMI and 17 UA patients. Based on the results of the baseline measurements, we decided to assess thrombin generation (1 pM TF +/- thrombomodulin), factor XIa and D-dimer measurements in the follow-up samples (Table 3, Figs 3 and 4).

Overall, the thrombin generation potential was enhanced during the acute event compared to 1 and 6 months afterwards (Fig 3). At the time of the acute thrombotic event, ACS patients had a significantly higher velocity index, increased peak height and ETP (all p<0.0001). Furthermore, there was a significantly diminished ETP reduction at baseline compared to 1 and 6 months follow-up (p<0.0001). Overall, we observed a decreased ETP reduction (<50%) during the first 6 months following a coronary event. There was no significant difference between the baseline and follow-up measurements in the time dependent thrombin generation parameters (Table 3, time to peak).

As depicted in Fig 4, no significant difference was observed between both D-dimer and factor XIa levels at the acute coronary event and 1 and 6 months afterwards. Different from the complete ACS group (baseline), median D-dimer levels at the three time points in the ACS follow-up group were below the cut-off value of 500 μg/L (Fig 4A). Factor XIa levels during follow-up showed a U-shape pattern, with a significant higher mean factor XIa level at 6 months compared to 1 month (p = 0.006) (Fig 4B). At all three time points the mean factor XIa plasma level was above the reference range. Different from the baseline measurements, no significant difference was observed in thrombin generation, D-dimer and factor XIa levels between the three ACS subgroups (STEMI, NSTEMI, UA) at 1 and 6 months (data not shown).

### Recurrent events

Clinical outcome was recorded at 1 and 6 months, which comprised the combined endpoint of cardiovascular death, recurrent MI or second coronary intervention. During the 6 months follow-up period, nine out of 73 patients suffered from a recurrent event (n = 2 cardiovascular mortality, n = 4 elective PCI, n = 2 myocardial infarction, n = 1 CABG). No significant associations were observed between the baseline thrombin generation parameters lag time, ETP, velocity index, time to peak, and ETP reduction, and recurrent cardiovascular events. However, the peak height was significantly associated with the risk of a recurrent event (OR: 4.9 (95%CI 1.2–20.9) for the highest quartile of peak thrombin generation (>177 nM). Furthermore, high factor XIa levels on admission predicted recurrent cardiovascular events with an OR of 4.5 (95%CI 1.1–18.9), whereas no associations were found for the antithrombin complexes and D-dimer levels.

## Discussion

In the present study we compared the coagulation profile of ACS patients to non-ACS patients. Overall, ACS patients had an enhanced prothrombotic phenotype as demonstrated by an increased thrombin generation potential with enhanced activation of the intrinsic pathway of coagulation and attenuated anticoagulant function of the protein C pathway. Furthermore, at the acute moment ACS patients had ongoing coagulation activity demonstrated by increased levels of circulating factor XIa and D-dimer. In accordance with the degree of coronary occlusion, STEMI patients had the highest prothrombotic profile. During follow-up thrombin generation was attenuated at 1 and 6 months, whereas D-dimer and factor XIa levels were not significantly different from baseline levels. Both, maximum thrombin generation and circulating factor XIa levels, predicted recurrent cardiovascular events with an odds ratio of approximately 5, suggesting potential clinical applicability of these laboratory assays.

Experimental data suggest that both factor XI and XII are involved in atherothrombosis. Using factor XI and XII null mice, it was shown that absence of one of the two proteins protects against carotid artery injury induced thrombosis [17]. The role of factor XI in experimental atherothrombosis was further demonstrated through antibody inhibition in both mice and baboon models [18]. Activation of factor XII and factor XI might result from components within the atherosclerotic lesion [19,20] or from activating platelets releasing polyphosphates [21]. Despite experimental evidence, limited clinical data on factor XIa and cardiovascular disease are available. Regarding factor XII, clinical studies on the relationship with coronary risk showed contradictory results [22–24]. However, for factor XI it was demonstrated by Minnema and colleagues that in patients with myocardial infarction factor XI activity was enhanced, as suggested by the presence of factor XIa-C1 inhibitor complexes in 24% of AMI patients and 8% of patients with unstable angina pectoris. In contrast, no increase in factor XIIa-inhibitor complexes was observed [25]. In another study it was shown that higher plasma levels of factor XI (XIc), but lower levels of factor XII (XIIc), were associated with increased risk for myocardial infarction in men [24]. Furthermore, detectable plasma factor XIa levels were demonstrated in 96% of ACS patients and 21% of stable coronary artery disease patients. In this study plasma factor XIa levels were assessed using a clotting assay, measuring the prolongation of the clotting time upon addition of an inhibitory factor XIa antibody [26]. In subsequent studies using this assay, quantifiable factor XI activity was demonstrated in patients with different cardiovascular diseases [27–29].

In the present study we observed increased plasma levels of factor XIa in ACS compared to non-ACS patients, which may mean that more factor XIa is generated during coronary occlusion or that there is a slower rate of factor XIa inhibition, allowing more of the factor XIa that is formed to circulate in its free form. Previously, we reported on the development of a new thrombin generation based factor XIa assay and the application of this method in plasma from patients with an acute myocardial infarction (AMI) [16]. In this prospective cohort study factor XIa levels were also significantly higher in AMI patients compared to healthy controls. Different from the present study, factor XIa levels in AMI patients were higher on admission compared to six months after the event. Also, factor XIa levels were not associated with recurrent cardiovascular events. However, there were some distinctions between the two study designs that might have contributed to the differences in results. First of all in this study the complete ACS spectrum was included, whereas the previous study only included first AMI patients. Other differences between the studies include the sample size, the time of blood drawing (emergency department versus ambulance) and the follow-up time (12 versus 6 months).

Regarding plasma thrombin generation, different studies observed that compared to healthy controls and patients with stable coronary artery disease, plasma thrombin generation was significantly elevated during an acute coronary event [30–34]. However, it remains difficult to determine whether increased coagulation is the cause or the consequence of a thrombotic event. Plaque rupture induces activation of coagulation, as was demonstrated in an experimental model [26]. Also, substantial evidence showed that coagulation enzymes such as thrombin contribute to progression of atherosclerosis, thereby potentially increasing the risk for atherothrombosis [7].

In a previous study of patients with an AMI, a trend of an inverse association of thrombin generation with recurrent cardiovascular events was demonstrated: patients with the lowest ETP values had the highest risk for recurrent events [32]. In a recent case-control study (the Glasgow MI Study) of patients diagnosed with AMI three to nine months prior to blood collection, plasma thrombin generation was significantly increased compared to controls [33]. A subsequent prospective study of this population (The Glasgow MONICA Study) observed that a significantly prolonged lag time and lower peak height and ETP were associated with first cardiovascular events. However, after adjustment for classical cardiovascular risk factors, these associations were no longer significant [unpublished data]. In the PROSPER Study, we also observed no overall association of thrombin generation parameters with incident coronary heart disease, except for a modest positive association of maximum thrombin generation [34]. What are possible explanations for these differences between previous studies and our current observation of a positive association between thrombin generation and cardiovascular events? First of all and as mentioned earlier, in the current study samples were collected at a very early stage after coronary occlusion occurred, i.e. during transportation to the hospital. Care was also taken to collect blood prior to administration of any antithrombotic medication. In contrast, most of the previous studies collected plasma months to years before or after the event. Furthermore, inclusion criteria differed between various studies and might contribute to differences in observed effects.

There were some limitations to the study. As discussed previously, the presence of four different inhibitors of factor XIa in plasma will influence the stability of factor XIa in samples [16]. The conclusion from our previous study that the time to processing should be standardized in order to minimize inactivation of the factor XIa protease is still valid. Although the exact time between blood collection and processing into plasma was not recorded, blood collection tubes were immediately transported to the laboratory for processing after arrival at the emergency department. A strong improvement of the current study design was the identical pre-analytical phase for ACS and non-ACS patients. Furthermore, the relatively high number of STEMI patients suggests a selection bias. Possible explanations are that the two cooperating hospitals in our study both have an interventional cardiology department, which might have resulted in the transportation of patients with a high STEMI suspicion to one of these hospitals, instead of to the other regional hospital, without an interventional cardiology department. Also, blood was drawn in the ambulance when the ambulance staff was transporting patients with chest pain suspect for ACS. It might be that blood was more frequently collected from STEMI patients, since they might have had a higher ACS suspicion. Next, we could speculate that patients with unstable angina compared to STEMI patients more frequently used self-transportation to the hospital. Regarding the recurrent events, a part consisted of patients with an elective PCI, which were all diagnosed with multiple vessel disease at the primary PCI. Based on their pre-existing more severe coronary heart disease, the elective PCI was probably triggered by a non-culprit lesion form the primary PCI, instead of a de novo recurrent event. Last, because of the relatively small number of patients and recurrent events, the data should be regarded as exploratory.

In conclusion, the potential to generate thrombin, D-dimer and circulating factor XIa levels were increased in ACS patients compared to patients without a cardiac event. Moreover, compared to clinical stabilization, thrombin generation was significantly enhanced during the acute thrombotic event. This study is the first to demonstrate the positive associations between both factor XI activity, thrombin generation, and recurrent cardiovascular events. Given the recent demonstration of the antithrombotic potential of specific anti-factor XI agents in patients at risk of venous thromboembolism [35], the future use of such agents in patients at risk of atherothrombosis also merits exploration.
